# Mapping gene and gene pathways associated with coronary artery disease: a CARDIoGRAM exome and multi-ancestry UK biobank analysis

**DOI:** 10.1038/s41598-021-95637-9

**Published:** 2021-08-12

**Authors:** Praveen Hariharan, Josée Dupuis

**Affiliations:** 1 MAASHA Trust, 154 E Central St #205, Natick, MA 01760 USA; 2 Department of Biostatistics, Boston University School of Public Health, Boston, MA USA

**Keywords:** Genetic databases, Cardiovascular genetics, Genetics research, Epidemiology, Risk factors, Cardiology

## Abstract

Coronary artery disease (CAD) genome-wide association studies typically focus on single nucleotide variants (SNVs), and many potentially associated SNVs fail to reach the GWAS significance threshold. We performed gene and pathway-based association (GBA) tests on publicly available Coronary ARtery DIsease Genome wide Replication and Meta-analysis consortium Exome (n = 120,575) and multi ancestry pan UK Biobank study (n = 442,574) summary data using versatile gene-based association study (VEGAS2) and Multi-marker analysis of genomic annotation (MAGMA) to identify novel genes and pathways associated with CAD. We included only exonic SNVs and excluded regulatory regions. VEGAS2 and MAGMA ranked genes and pathways based on aggregated SNV test statistics. We used Bonferroni corrected gene and pathway significance threshold at 3.0 × 10^–6^ and 1.0 × 10^–5^, respectively. We also report the top one percent of ranked genes and pathways. We identified 17 top enriched genes with four genes (**PCSK9, FAM177, LPL, ARGEF26),** reaching statistical significance (p ≤ 3.0 × 10^–6^) using both GBA tests in two GWAS studies. In addition, our analyses identified ten genes (DUSP13, KCNJ11, CD300LF/RAB37, SLCO1B1, LRRFIP1, QSER1, UBR2, MOB3C, MST1R, and ABCC8) with previously unreported associations with CAD, although none of the single SNV associations within the genes were genome-wide significant. Among the top 1% non-lipid pathways, we detected pathways regulating coagulation, inflammation, neuronal aging, and wound healing.

## Introduction

Coronary artery disease (CAD) is a complex disease phenotype influenced by numerous genotypic (polygenic) and environmental factors^1–3^. While much work to understand the effect of various environmental factors was undertaken in the past century, recent genome-wide association studies (GWAS) have identified multiple genetic loci associated with CAD^4,5^.

Although GWAS has identified more than 160 loci with one or more Single Nucleotide Variants (SNVs) significantly associated with CAD risk, many are in non-coding and intergenic regions with unknown functional significance^3,6^. Many associated SNVs are in linkage disequilibrium (LD) with existing genetic variants, have pleiotropic effects, and are involved in overlapping pathways^6,7^. The causal pathways of these pleiotropic genetic variants with CAD have yet to be elucidated. A recent study by Coronary ARtery DIsease Genome wide Replication and Meta-analysis (CARDIoGRAM) consortium investigators confirmed isoforms of the LPA gene (Lipoprotein-A), and the PCSK9 (Proprotein convertase subtilisin/kexin type) gene and discovered new rare isoforms of ANGPTL4 (Angiopoietin-like 4) gene linked with lipid homeostasis to be associated with CAD^1^. However, the common and rare SNVs only contribute to a portion of the heritability of CAD^8^. Moreover, reporting only the top associated GWAS SNVs can have many limitations. First, it can shift the focus to a narrow set of gene-associated pathways to explain complex disease phenotypes. For example, top SNV based GWAS studies in CAD have mainly identified SNVs associated with lipid, blood pressure, and obesity homeostasis^1^. Second, it can lead to omitting SNVs that fail to reach the GWAS significance threshold yet are involved in disease pathophysiology. Third, the addition of SNV-based genetic scores to traditional risk factors only moderately improved discriminant statistics for CAD prediction^3,9^. Rather than focusing on a few SNVs strongly associated with CAD, by considering multiple SNVs in a gene and multiple genes in a pathway, our ability to identify novel genes and causal pathways can improve^10–13^. This premise is the basis of many gene-based association analyses (GBA), which investigates the association of phenotypes with a group of markers (usually SNVs) within a gene rather than most-associated individual markers. In GBA, a gene association statistic is calculated using individual SNV association statistics after assigning SNVs to genes based on a-priori criteria. A permutation (gene-based or phenotype-based) or resampling approach is often used to correct for gene characteristics such as LD structure and size. Often GBA forms the basis for gene pathway association analysis (GPA), which tests the association of biologically related genes in a predefined pathway with the phenotype of interest using a self-contained or competitive null hypothesis^14^. While the self-contained test assumes the null-hypothesis that none of the genes are associated with the phenotype, the competitive test assumes a group of genes in a pathway no more likely to be associated with the phenotype than other genes^10,14^. Both GBA and GPA can put into perspective and supplement the individual SNVs identified through GWAS, especially for complex phenotypes influenced by polygenicity^15^. For instance, the multiple SNVs associated with human height identified through GWAS were put into perspective using GBA and GPA as being located in genes within Hedgehog, Transcription Growth Factor-Beta, and growth hormone pathways that affect skeletal growth^13^. Investigating multiple causal pathways can help identify biomarkers and therapeutic agents with pleiotropic effects beyond what can be achieved by focusing on single gene coding variants, like PCSK9^1,9^.

Many tools can perform GBA and GPA including Multi-marker analysis of genomic annotation (MAGMA) or Versatile gene-based association study (VEGAS2)^10,15,16^. Each tool is unique and differs in the type of input data used, type of annotation used, type of null hypothesis used, the methodology of assigning gene and pathway scores, type of approach used (permutation or resampling), and the type of software used (proprietary or open-source)^10^. While there is a lack of consensus on the superiority of a particular tool, tools using competitive null hypothesis generally take into account heritability and genomic inflation. They are more suitable for testing multiple genes and pathways using GWAS results.^10,15,17^. Recent methodological reviews using simulated and real GWAS summary statistics have reported that VEGAS, MAGMA, and GSEA are the most popular and powerful GBA and GSA tools^16,17^. Wojick et al. compared 21 different methods using WTCCC (Wellcome Trust Case Control Consortium) data and concluded VEGAS2 had the highest specificity in GBA^16^. Both VEGAS and MAGMA use a competitive null hypothesis, are available open-source, and use GWAS summary statistics as input data^18,19^. Hence, this study aims to identify genes associated with CAD using results from non-synonymous autosomal genetic variants in the CARDIoGRAM Exome studies (CGEX) with VEGAS2 and further compare with MAGMA^19^. We further aimed to compare our results in an independent multi-ancestry Pan-UK biobank (PUBB) GWAS study^20^. Given the limitations of reporting only the top associated SNVs for a complex disease phenotype like CAD, we further aimed to map multiple gene pathways associated with CAD, in particular assess the contribution of non-lipid based pathways.

## Results

**Figure 1:**
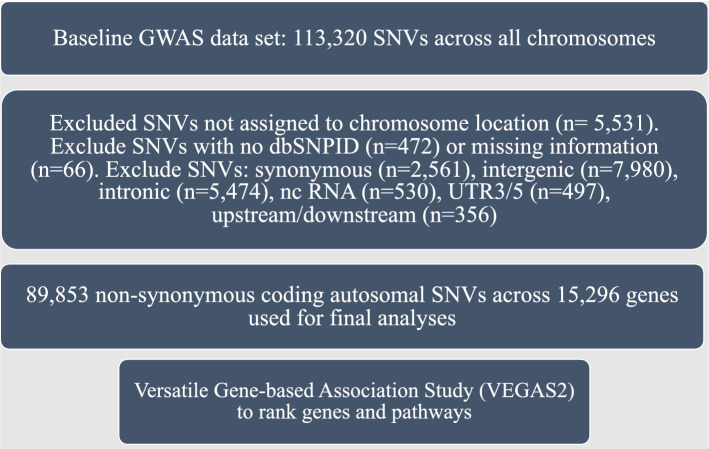
SNVs and corresponding genes used in final analysis (42,335 cases and 78,240 controls). *SNVs* single nucleotide variants, *GWAS* genome wide association studies, *RNA* ribonucleic acid, *UTR* untranslated region.

We included 89,853 non-synonymous coding SNVs from the CGEX study across 22 autosomes in our final analysis (Fig. 1). We did not find evidence of systemic inflation of p-values in the QQ plot analysis (λ GC = 1.06); however, when restricted to rare variants, we observed some evidence of inflation in p values (λ GC = 1.29, Supplement 1 FI). In a prior report, the CGEX investigators adjusted test statistics for GC before performing GWAS association and included a homogenous population (Supplement 1 (I), Western European ancestry). Hence, we did not perform a second GC correction of GWAS association statistics after meta-analysis as it may not represent overdispersion due to population stratification but rather represent true genetic signals^21,22^.

The PUBB study, our replication cohort, included 85,206 SNVs with MAF ≥ 0.1% across 442,574 multi-ancestry individuals in our final analysis. We did not find any evidence of overdispersion of association statistics (λ GC = 1.11), even when restricting to rare variants (λ GC = 1.09, MAF ≤ 5%, Supplement 2 FI).

The manhattan plot in Supplement 1 (FII) and Supplement 2 (FII) highlights SNV association results across all autosomes in the CGEX and PUBB study.

### VEGAS 2 GBA

**Figure 2:**
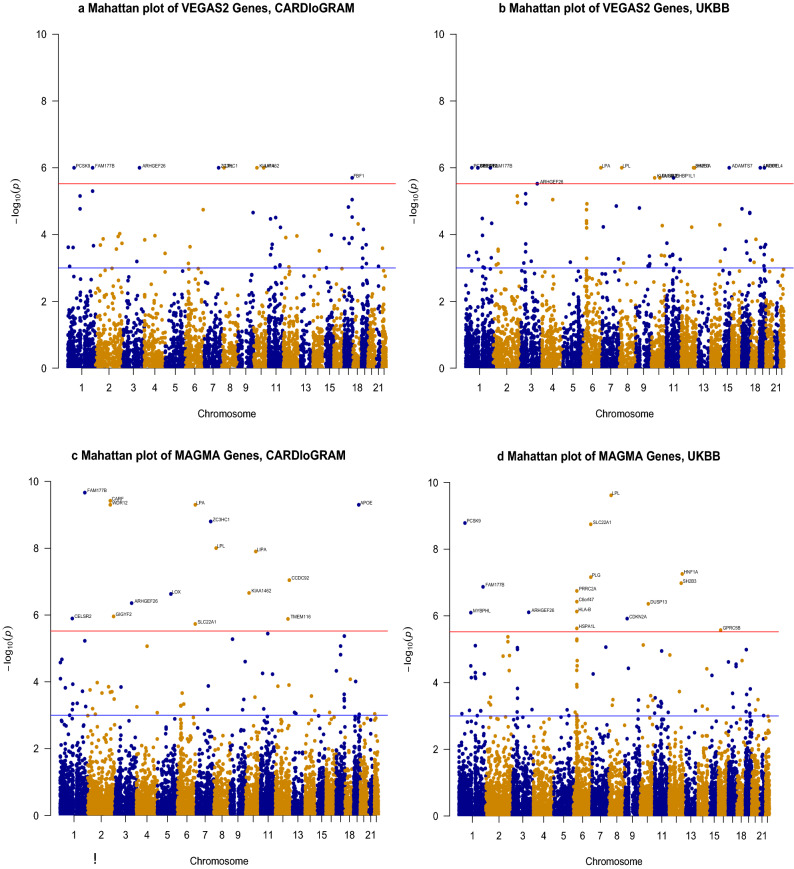
Manhattan Plots of VEGAS2 and MAGMA gene-based association analysis.

Table 1 lists the top one percent CAD-associated genes identified by VEGAS GBA in the CGEX study and the PUBB study. Supplement 1 (V) describes the entire list of CAD-associated genes identified by VEGAS2 GBA in the CGEX study. Based on VEGAS2 GBA, most of the genes identified in the top one percent of the associated list carried at least one SNV meeting a significance threshold of p ≤ 1 × 10^–4^. VEGAS2 GBA in CGEX identified multiple enriched genes (KIAA1462/JCAD, LIPA, FAM177B, PCSK9, ARHGEF26, ZC3HC1, LPL, FBF1) that met our significance threshold (p ≤ 3 × 10^–6^) and further confirmed them in PUBB (Table 1). Many genes have been previously reported to be associated with CAD by a different GBA methods (Table 1 and Supplement 1, VII)^23,24^. In addition, we further identified nine significant genes in PUBB (ADAMTS7, APOE, LPA, SH2B3, HNF1A, CELSR2, MYBPHL, ANGPTL4, EHBP1L1) that have been previously reported to be associated CAD by GBA (Table 1)^6,23,24^. The Manhattan plots (Fig. 2a,b) describes the list of genes across all autosomes in the CGEX and PUBB study.

**Table 1:** Top enriched genes identified using VEGAS2, and MAGMA GBA in CARDIoGRAM and Pan UK Biobank (UKBB).

Top significant genes (p ≤ 3 × 10^–6^) identified using VEGAS2, and MAGMA GBA in either CARDIoGRAM or UKBB							
VEGAS2 GBA				MAGMA GBA			
Cardiogram	P	UKBB	P	CARDIOGRAM	P	UKBB	P
*KIAA1462*^*†*^	**1.0 × 10**^**–6**^	ADAMTS7*	**1.0 × 10**^**–6**^	*PCSK9*^*‡*^	**1.7 × 10**^**–11**^	ADAMTS7*	**7.8 × 10**^**–16**^
LIPA*	**1.0 × 10**^**–6**^	APOE*	**1.0 × 10**^**–6**^	FAM177B*	**2.2 × 10**^**–10**^	LPA*	**4.7 × 10**^**–15**^
FAM177B*	**1.0 × 10**^**–6**^	LPA*	**1.0 × 10**^**–6**^	CARF*	**3.8 × 10**^**–10**^	APOE*	**7.3 × 10**^**–13**^
*PCSK9*^*‡*^	**1.0 × 10**^**–6**^	*SH2B3*^*‡*^	**1.0 × 10**^**–6**^	WDR12^‡^	**5.0 × 10**^**–10**^	*LPL*^*†*^	**2.4 × 10**^**–10**^
*ARHGEF26*^*‡*^	**1.0 × 10**^**–6**^	*LPL*^*†*^	**1.0 × 10**^**–6**^	LPA*	**5.0 × 10**^**–10**^	*PCSK9*^*‡*^	**1.7 × 10**^**–9**^
*ZC3HC1*^*§*^	**1.0 × 10**^**–6**^	*PCSK9*^*‡*^	**1.0 × 10**^**–6**^	APOE*	**5.0 × 10**^**–10**^	SLC22A1	**1.8 × 10**^**–9**^
*LPL*^*†*^	**1.0 × 10**^**–6**^	*HNF1A*^*‡*^	**1.0 × 10**^**–6**^	*ZC3HC1*^*§*^	**1.6 × 10**^**–9**^	*HNF1A*^*‡*^	**5.6 × 10**^**–8**^
FBF1*	**2.0 × 10**^**–6**^	FAM177B*	**1.0 × 10**^**–6**^	*LPL*^*†*^	**9.9 × 10**^**–9**^	PLG^†^	**6.9 × 10**^**–8**^
		*CELSR2*^*§*^	**1.0 × 10**^**–6**^	LIPA*	**1.3 × 10**^**–8**^	SH2B3^‡^	**1.1 × 10**^**–7**^
		MYBPHL*	**1.0 × 10**^**–6**^	CCDC92*	**9.0 × 10**^**–8**^	FAM177B*	**1.4 × 10**^**–7**^
		ANGPTL4*	**1.0 × 10**^**–6**^	*KIAA1462*^*†*^	**2.2 × 10**^**–7**^	PRRC2A	**1.8 × 10**^**–7**^
		DUSP13	**2.0 × 10**^**–6**^	LOX*	**2.3 × 10**^**–7**^	C6orf47*	**3.8 × 10**^**–7**^
		EHBP1L1^§^	**2.0 × 10**^**–6**^	*ARHGEF26*^*‡*^	**4.4 × 10**^**–7**^	DUSP13	**4.4 × 10**^**–7**^
		*KIAA1462*^*†*^	**2.0 × 10**^**–6**^	GIGYF2^‡^	**1.1 × 10**^**–6**^	HLA-B	**7.4 × 10**^**–7**^
		*ARHGEF26*^*‡*^	**3.0 × 10**^**–6**^	*CELSR2*^*§*^	**1.3 × 10**^**–6**^	*ARHGEF26*^*‡*^	**7.8 × 10**^**–7**^
				TMEM116*	**1.3 × 10**^**–6**^	MYBPHL*	**8.0 × 10**^**–7**^
				SLC22A1*	**1.9 × 10**^**–6**^	CDKN2A*	**1.2 × 10**^**–6**^
						HSPA1L*	**2.4 × 10**^**–6**^
						GPRC5B	**2.7 × 10**^**–6**^
**Top one percent genes identified using VEGAS2, and MAGMA GBA in both CARDIoGRAM and UKBB with p ≥ 3 × 10**^**–6**^							
*CELSR2*^*§*^	7.0 × 10^–6^	DHX58*	1.6 × 10^–4^	MYBPHL*	6.8 × 10^–4^	DHX58*	2.1 × 10^–4^
MYBPHL*	1.7 × 10^–5^	*LIPG*^*†*^	1.4 × 10^–4^	DHX58*	1.5 × 10^–5^	*LIPG*^*†*^	2.2 × 10^–5^
LPA*	1.8 × 10^–5^	TNS1*	1.1 × 10^–5^	*LIPG*^*†*^	8.4 × 10^–5^	TNS1*	1.6 × 10^–5^
DHX58*	1.5 × 10^–5^	*RAB37*	2.2 × 10^–5^	TNS1*	2.0 × 10^–4^	*RAB37*	3.3 × 10^–5^
*LIPG*^*†*^	4.8 × 10^–5^	*CD300LF*	2.3 × 10^–5^	*RAB37*	3.2 × 10^–4^	*CD300LF*	2.8 × 10^–6^
TNS1*	9.4 × 10^–5^	*QSER1*^*§*^	4.5 × 10^–4^	*CD300LF*	2.4 × 10^–4^	*SMG6*^*‡*^	2.4 × 10^–5^
*RAB37*	1.2 × 10^–4^	*ZC3HC1*^*§*^	1.4 × 10^–5^	*SMG6*^*‡*^	4.7 × 10^–5^	*QSER1*^*§*^	5.1 × 10^–4^
*CD300LF*	1.2 × 10^–4^	*SMG6*^*‡*^	1.7 × 10^–5^	*HNF1A*^*‡*^	1.2 × 10^–4^	*KIAA1462*^*†*^	7.5 × 10^–6^
*SMG6*^*‡*^	1.3 × 10^–4^			*QSER1*^*§*^	1.5 × 10^–3^	*CELSR2*^*§*^	3.2 × 10^–5^
*HNF1A*^*‡*^	1.2 × 10^–4^					*ZC3HC1*^*§*^	8.7 × 10^–6^
*QSER1*^*§*^	2.0 × 10^–4^						

Top one percent of the ranked genes in either VEGAS2 or MAGMA GBA are defined as top enriched genes.

P values of significant genes (p ≤ 3 × 10^–6^) highlighted in bold. Genes highlighted in italics were identified using VEGAS2, and MAGMA GBA in CARDIoGRAM and UKBB (Pan UK Biobank).

*Previously reported by Svishcheva et al.^23^.

^†^SNVs in corresponding genes previously reported by Van der Hast et al.^6^.

^‡^SNVs in corresponding genes previously reported by Nelson et al.^29^.

^§^SNVs in corresponding genes previously reported by Hartiala et al.^21^.

In the top one percent of CGEX associated genes above the Bonferroni-corrected threshold (p > 3 × 10^–6^) identified by VEGAS2, we identified 13 genes (**CELSR2**, KCNJ11, **HNF1A**, **ARHGAP25**, LRRFIP1, QSER1, ABCC8, UBR2, **ARVCF**, **TFPI**, MOB3C, **BDNF**, **CYP4V2**) without a single SNV meeting a significance threshold of p ≤ 1 × 10^–4^. Among these 13 genes, 7 (bolded) have been previously reported to be associated with CAD^5,6,8,25^. These results suggest the complementary role of GBA to identify significant loci and genes in addition to traditional GWAS studies^11^. Also, we identified the DUSP13 gene significantly associated (p = 2 × 10^–6^) with IHD in the PUBB study, which to our knowledge, has not been previously reported to be associated with CAD. However, DUSP13 gene failed to reach statistical significance (p = 0.17) in the CGEX study. When we performed a combined metanalysis (CGEX and PUBB GWAS) of DUSP13 gene p values using the fisher method, we obtained a gene-level p-value of 5.4 × 10^–6^. Of note, the FAM177B and the DUSP13 gene have also been linked to neurocognitive health and self-reported educational attainment, respectively^26,27^.

In addition, in the CGEX VEGAS2 enriched genes, we also identified KCNJ11, CD300LF/RAB37, SLCO1B1, LRRFIP1, QSER1, UBR2, MOB3C, and ABCC8 genes, that to our knowledge have not been previously described to be associated with CAD using GBA (Table 2). We would also like to highlight the previously unreported MST1R gene identified in the top 1% of MAGMA (p = 2.9 × 10^–4^) and VEGAS2 (Emp p = 1.9 × 10^–4^) in the PUBB study that was also identified in the CGEX study. The non-synonymous SNV in MST1R gene (rs2230590) was one of the top loci associated with intelligence and neurocognition by an independent international GWAS consortium^28^. We did not identify any previously reported CAD-associated SNVs in high LD (r^2^ > 0.8) with our SNVs in the enriched genes in (Table 2 and Supplement 2, VIII).

**Table 2:** Top enriched GBA genes previously unreported CAD associations.

Name	SNV in gene	Allele1/Allele2	Allele 1 frequency (%)	OR (CGEX, SNV p value)	OR (PUBB, SNV p value)	Amino-acid substitution	SIFT score (threshold ≤ 0.05)	Gene trait°
**FAM177B**	rs2378607	T/G	31%	1.06 (p = 1.1 × 10^–9^)	1.04 (p = 1.5 × 10^–7^)	[ATT] Ile3Ser[AGT]	0.07	Neurocognitive health
**FAM177B**	rs6683071	A/G	19%	1.07 (p = 1.2 × 10^–8^)	1.04 (p = 1.5 × 10^–5^)	[CAA]Gln143Arg[CGA]	0.99	Neurocognitive health
MST1R	rs2230590	T/C	49%	1.03 (p = 3 × 10^–3^)	1.03 (p = 2 × 10^–3^)	[CAA]Gln523Arg[CGA] ^‡^	1	Intelligence/BMI
PLCB3	rs12146487	A/G	17%	0.95 (p = 4 × 10^–5^)	0.97 (p = 2 × 10^–2^)	[CGC]Arg483His[CAC]	0.04	Obesity/HDL
KCNJ11	rs5215	C/T	37%	1.03 (p = 1.9 × 10^–4^)	1.004 (p = 6.2 × 10^–1^)	[GTC]Val250Ile[ATC] ^‡^	0.137	Diabetes, Benign islet cell hyperplasia
ANKLE1	rs77683348	A/G	3%	0.89 (p = 8.1 × 10^–5^)	1.03 (p = 2.3 × 10^–1^)	[CGG]Arg548Gln[CAG]	0	Breast/ovary cancer
SLCO1B1	rs4149056	C/T	17%	0.95 (p = 7.0 × 10^–5^)	0.99 (p = 4.6 × 10^–1^)	[GTG]Val174Ala[GCG]	NA	Statin response, serum metabolite levels, bilirubin and thyroxin levels
CD300LF/ RAB37	rs35489971	A/G	19%	1.05 (p = 6.4 × 10^–5^)	0.96 (p = 1.8 × 10^–5^)	[GTC]Val19Asp[GAC] ^‡^	0	C reactive protein, Fibrinogen levels
LRRFIP1	rs11680012	C/G	5%	1.14 (p = 1.8 × 10^–3^)	1.03 (p = 1.1 × 10^–1^)	[AGG]Arg634Thr[ACG]	0.47	Adiposity
QSER1	rs62618693	T/C	4%	0.92 (p = 1.9 × 10^–4^)	0.93 (p = 3 × 10^–4^)	[CGC]Arg1230Cys[TGC]	0.04	Type II Diabetes, Smoking
UBR2	rs62414610	A/G	4%	1.09 (p = 5.9 × 10^–4^)	1.04 (p = 3 × 10^–3^)	[GAG]Glu126Lys[AAG]	0.04	Obesity, and Lung cancer
MOB3C	rs6671527	A/G	47%	0.96 (p = 1.5 × 10^–4^)	0.98 (p = 1 × 10^–2^)	[CGA]Arg24Stop[TGA] ^‡^	NA	NA
ABCC8	rs757110	C/A	37%	1.03 (p = 9.5 × 10^–4^)	1.003 (p = 6.7 × 10^–1^)	[GCC]Ala1370Thr[ACC] ^‡^	0.29	Diabetes
**DUSP13**	rs6480771	T/C	43%	1.01 (p = 2.1 × 10^–1^)	1.04 (p = 3.9 × 10^–7^)	([AGC]Ser160Cys[GGC])	0.4	Neurocognitive health

Genes in bold with p values less than the Bonferroni-corrected significance threshold in CGEX (CARDIoGRAM) or PUBB (Pan UK Biobank) study (p ≤ 3 × 10^–6^).

SIFT score: Sorting Intolerant from Tolerant score. A score ranges from 0–1, and score ≤ 0.05 is suggestive to functional consequence from protein alteration based on amino acid sequence change.

*HDL* high density lipoprotein, *BMI* body mass index, *NA* not available, *OR* odds ratio for coronary artery disease.

^‡^Negative strand.

°Gene trait: Clinical phenotypes (maximum 2) obtained using the GWAS catalog (https://www.ebi.ac.uk/gwas/genes), the Ensembl genome database (https://www.ensembl.org), the National Center for Biotechnology ClinVar web-based database (https://www.ncbi.nlm.nih.gov/clinvar/variation/), and through pubmed search (https://pubmed.ncbi.nlm.nih.gov).

### MAGMA GBA

We identified many significant (p ≤ 3 × 10^–6^) genes (PCSK9, FAM177, CARF, WDR12, LPA, APOE, ZC3HC1, LPL, LIPA, CCDC92, KIAA1462, LOX, ARHGEF26, GIGYF2, CELSR2, TMEM116, SLC22A1) in the CGEX study using MAGMA GBA (Table 1). The Manhattan plots (Fig. 2c,d) describes the list of genes across all autosomes in the CGEX and PUBB study. All of the genes have been previously reported in CGEX and PUBB GWAS using a different type of GBA (Table 1)^23^. We further confirmed six genes (**PCSK9**, **FAM177**, LPA, APOE, **LPL**, **ARGEF26**) in the PUBB study (Table 1). Four of the six genes (highlighted) were identified as significant (p ≤ 3 × 10^–6^) by both GBA methods and in both CGEX and PUBB GWAS (Table 3).

**Table 3:** Top 17 enriched genes identified in both GBA methods across CARDIoGRAM (CGEX) and Pan UK Biobank (PUBB).

Name	Top SNV in gene (CGEX)	Top SNV in gene (PUBB)	Gene trait°
**FAM177B**	rs2378607	rs2378607	Neurocognitive health
**PCSK9**	rs11591147	rs11591147	Coronary artery disease, low density lipoprotein levels
**LPL**	rs328	rs328	Coronary artery disease, metabolic syndrome
**ARHGEF26**	rs12493885	rs12497267	Coronary artery disease, systolic blood pressure
KIAA1462/JCAD	rs3739998	rs3739998	Coronary artery disease, platelet count
ZC3HC1	rs11556924	rs11556924	Coronary artery disease, platelet count
LPA	rs41272114	rs3124784	Coronary artery disease, peripheral artery disease
DHX58	rs2074158	rs34891485	Coronary artery disease, high density lipoprotein levels
LIPG	rs2000813	rs77960347	Total cholesterol levels, high density lipoprotein levels
MYBPHL	rs629001	rs629001	PR interval/intelligence, tau protein levels
CELSR2	rs72703203	rs72703203	Coronary artery disease, low density lipoprotein levels
TNS1	rs918949	rs918949	Coronary artery disease, blood pressure
SMG6	rs903160	rs903160	Coronary artery disease, body mass index
HNF1A	rs1169288	rs1169288	Coronary artery disease, C reactive protein
CD300LF	rs35489971	rs35489971	C reactive protein, fibrinogen levels
RAB37	rs35489971	rs35489971	C reactive protein, fibrinogen levels
QSER1	rs62618693	rs62618693	Type II diabetes, smoking

Genes highlighted in bold with p values less than the Bonferroni-corrected significance threshold (p ≤ 3 × 10^–6^) in both CGEX and PUBB GWAS.

°Gene trait: clinical phenotypes (maximum 2) obtained using the GWAS catalog (https://www.ebi.ac.uk/gwas/genes), the Ensembl genome database (https://www.ensembl.org), the National Center for Biotechnology ClinVar web-based database (https://www.ncbi.nlm.nih.gov/clinvar/variation/), and through pubmed search (https://pubmed.ncbi.nlm.nih.gov).

In addition, we identified 17 genes among the top one percent of ranked VEGAS2 and MAGMA genes that were identical in CGEX and UKBB (Fig. 4, Table 3). SNVs in many of the genes listed in Table 3 have been previously reported to be associated with CAD.

**Figure 3:**
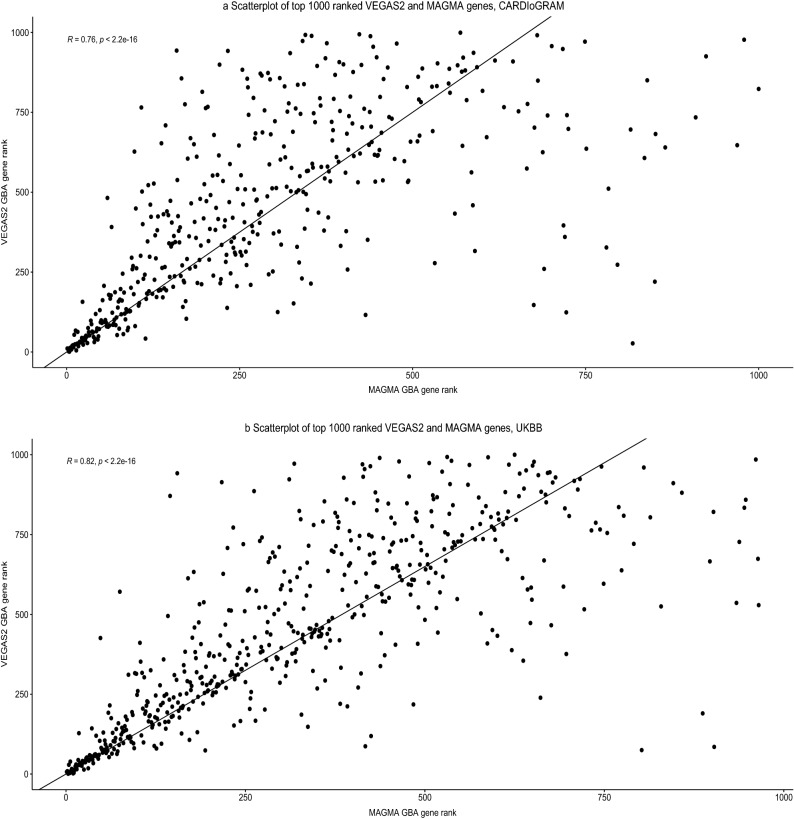
Scatter plot of VEGAS2 and MAGMA ranked genes. *R* Spearman correlation coefficient, *GBA* gene based association analysis.

**Figure 4:**
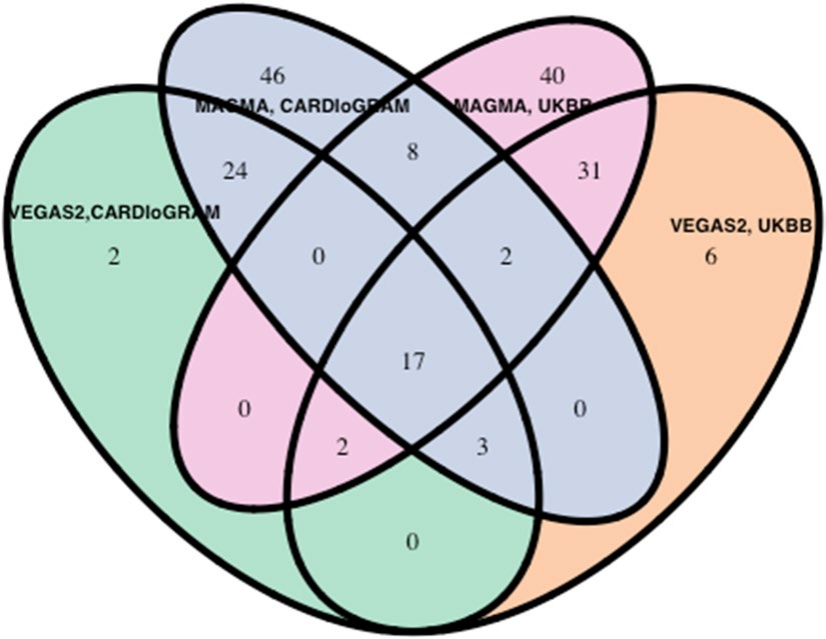
Venn diagram of top one percent VEGAS2, MAGMA ranked genes in CARDIoGRAM and Pan UK Biobank study. *UKBB* United Kingdom Biobank.

There was a significant correlation between ranks of the genes using VEGAS2 GBA and MAGMA GBA in CGEX (Spearman correlation r = 0.76, p < 2.2 × 10^–16^) and PUBB (Spearman correlation r = 0.82, p < 2.2 × 10^–16^) study (Fig. 3). Among the top one percent of VEGAS2 GBA genes, almost 94% (44/47) of genes in the CGEX study and 85% (52/61) genes in the PUBB study were confirmed in the top one percent of the MAGMA GBA gene list, respectively (Fig. 4). Figure 3a,b demonstrate the correlation of ranking between VEGAS2 and MAGMA genes, with a high concordance of ranks noted in the top 1% genes.

### VEGAS2 GPA

Supplements 1 (VI) and 2 (V) describe the list of pathways identified by VEGAS2 GPA in the CGEX and PUBB study. We identified multiple lipid homeostasis and lipoprotein metabolism pathways in the top enriched pathways, confirming their role in CAD pathophysiology (Table 4). We were also able to identify other critical regulatory pathways of coagulation (Panther_Blood_coagulation, pathway, Emp p = 8.0 × 10^–6^), inflammation (PID_AMB2_NEUTROPHILS_PATHWAY, Emp p = 9.2 × 10^–6^), neuronal aging (GO:0043523_regulation_of_neuron_apoptosis, Emp p = 2.2 × 10^–4^), and wound healing (GO:0042060_wound_healing, pathway Emp p = 4.1 × 10^–4^) among the top one percent in the CGEX. We found similar pathways modulating inflammation, and neurologic aging among the top one percent of pathways in the PUBB study. However, only the coagulation pathway met the Bonferroni corrected significance threshold (P ≤ 1 × 10^–5^, Table 4). This information provides further evidence of the complex pathophysiology of CAD that would have otherwise received less attention based only on top SNVs^12^.

**Table 4:** Top one percent VEGAS2 lipid and non-lipid based enriched pathways in CARDIoGRAM (CGEX)  and Pan UK Biobank (PUBB).

Molecular pathway name	Rank	Genes in the pathway	Empirical P value	Rank	Genes in the pathway	Empirical p value (P)
	CARDIoGRAM			PUBB		
**Lipid pathway**						
**GO:0055088_lipid_homeostasis**	1	16	**1.0 × 10**^–6^*	12	18	**8.0 × 10**^–6^*
**GO:0016298_lipase_activity**	2	32	**2.0 × 10**^–6^*	76	36	7.0 × 10^–4^
**REACTOME_LIPID_DIGESTION_MOBILIZATION_AND_TRANSPORT**	7	15	**6.0 × 10**^–6^*	4	17	**2.0 × 10**^–6^*
**GO:0034368_protein-lipid_complex_remodeling**	5	8	**4.0 × 10**^–6^*	8	10	**2.0 × 10**^–6^*
**Coagulation**						
**PANTHER_BIOLOGICAL_PROCESS_Blood_clotting**	8	14	**8.0 × 10**^–6^*	38	29	1.9 × 10^–4^
**Inflammation**						
PID_AMB2_NEUTROPHILS_PATHWAY	26	10	9.2 × 10^–5^	42	10	9.2 × 10^–5^
GO:0042060_wound_healing	47	36	4.2 × 10^–4^	882	51	7.0 × 10^–2^
**Neuronal aging**						
GO:0043523_regulation_of_neuron_apoptosis	42	13	2.2 × 10^–4^	123	15	1.9 × 10^–3^
GO:0031175_neurite_development	314	47	1.9 × 10^–2^	51	55	3.2 × 10^–4^

*GO* gene ontology, *PID* pathway interaction database, *PUBB* Pan UK Biobank.

Empirical p value: Obtained from resampling genes from the pathway and calculated as the number of instances the summed chi-squared test statistics per resample exceeds observed test statistic.

*Pathways in bold with empirical pathway significance threshold was defined was p ≤ 10^–5^.

**Table 5:** Top one percent MAGMA lipid and non-lipid based enriched pathways in CARDIoGRAM (CGEX) or Pan UK Biobank (PUBB).

Molecular pathway name	Rank	Genes in the Pathway	P value	Rank	Genes in the pathway	P value
	CARDIoGRAM			PUBB		
**Lipid pathway**						
**GO:0017127_cholesterol_transporter_activity**	2	10	**5.9 × 10**^–8^*	4	9	**1.5 × 10**^–7^*
**GO:0034368_protein-lipid_complex_remodeling**	3	14	**2.1 × 10**^–7^*	5	14	**1.6 × 10**^–7^*
**GO:0055088_lipid_homeostasis**	13	67	3.9 × 10^–5^	13	69	**1.2 × 10**^–6^*
**Coagulation**						
GO:0050817_coagulation	122	265	3.1 × 10^–3^	85	263	9.1 × 10^–4^
**Inflammation**						
amb2_neutrophils_pathway_amb2_Integrin_signaling	15	28	3.9 × 10^–5^	38	27	8.7 × 10^–5^
GO:0042060_wound_healing	69	360	9.6 × 10^–4^	78	356	7.7 × 10^–4^
**Neuronal aging**						
GO:0048156_tau_protein_binding	193	5	5.8 × 10^–3^	42	3	1.2 × 10^–4^
GO:0043523_regulation_of_neuron_apoptosis	666	84	3.3 × 10^–2^	591	81	3.0 × 10^–2^

*GO* gene ontology, *PUBB* Pan UK Biobank.

*Pathways in bold with empirical pathway significance threshold was defined was p ≤ 5 × 10^–6^.

### MAGMA GPA

Supplements 1 (X) and 2 (VII) describe the list of pathways identified by MAGMA GPA in the CGEX and PUBB study. While we could not perform a head-to-head comparison with the VEGAS2 pathway analysis, we found a very similar category of pathways within the top one percent of MAGMA pathways compared to VEGAS2 pathways (Tables 4 and 5). In addition to the highly significant lipid pathways, we identified regulating coagulation (GO:0050817_coagulation), inflammation (amb2_neutrophils_pathway_amb2_Integrin_signaling), neuronal aging (GO:0048156_tau_protein_binding), and wound healing (GO:0042060_wound_healing) (Table 5). These findings further consolidate the complex pathophysiology of CAD demonstrated by VEGAS2 and MAGMA GPA.

## Discussion

In this study, through VEGAS2 GBA and GPA, we identified multiple genes regulating coagulation, inflammation, wound healing, and neuronal aging pathways to be associated with CAD in the CGEX study. We further confirmed many of these findings in an independent large multi-ancestry PUBB study. We were also able to replicate our results using a MAGMA GBA and GPA in CGEX and PUBB. We identified 17 top enriched genes with four genes reaching statistical significance (p ≤ 3 × 10^–6^) using both GBA methods in two GWAS studies. SNVs in many of these genes have been reported to be associated with CAD using single SNV association analyses^6,8,21,29,30^. In addition, we identified few genes that have not been previously reported to be associated with CAD. VEGAS2 and MAGMA GBA further identified multiple genes carrying sub GWAS threshold SNVs and pathways associated with CAD.

While multiple single SNV GWAS analyses using CGEX and PUBB data have been published, few investigations have focused on GBA and GPA (Table 3). VEGAS2 and MAGMA GBA identified FAM177B as a significant gene in both CGEX and PUBB, in addition to PCSK9, LPL, and ARGEF26 (Tables 2, 3). More recently, loci in the FAM177B gene have been identified by gene-based association analyses in the UK Biobank cohort by Svishcheva et al.^23^ The FAM177B (rs6683071, A/G, Allele 1 = 19%) missense variant has been predicted to yield protein FAM177B isoform X1 ([CAA]Gln143Arg[CGA]). The FAM177B isoform X1 (rs6683071, OR = 1.07), and FAM177B isoform X2 (rs2378607, OR = 1.06) have been predicted by the Sorting Intolerant from Tolerant (SIFT) tool as tolerant missense variants. However, the isoform X2 has been reported to have a higher probability of affecting protein function based on a lower SIFT score (SIFT score = 0.07). We did not observe high LD between the two SNVs (r^2^ = 0.48). FAM177B contained the highest-ranked non-synonymous coding SNV (p = 4.2 × 10^–6^) associated with one of the cognition phenotypes in the CANTAB study though it failed to reach genome-wide significance^26^. FAM177B conferred an increased risk of CAD in our study.

Among other SNVs, we identified loci in MYBPHL and DUSP13 gene in CGEX and PUBB. MYBPHL has been previously associated with PR-interval, intelligence, neurocognitive function, tau protein levels, and self-reported educational attainment^27,28,31,32^. At the same time, MYBPHL (rs629001, OR = 1.08, p = 2.1 × 10^–5^ (CGEX), p = 7.6 × 10^–7^ (PUBB)) increased the risk of CAD in our study. SNV (rs629001) in the MYBPHL gene has been reported as a non-synonymous coding variant by the Leducq Consortium CADGenomics investigators associated with CAD^33^. MYBPHL is myofilament protein overexpressed in human atrial tissue and its concentration increases in the serum after cryoablation or radiofrequency ablation induced atrial damage^34^. MYBPHL is also located in chromosome 1p13.3, which hosts other high-risk CAD loci^35^. We did not identify any previously reported CAD-associated SNVs in high LD (r^2^ > 0.8) with our SNVs in DUSP13 or MYBPHL. DUSP13 gene (rs6480771, OR = 1.04, p = 3.9 × 10^–7^ (PUBB), p = 0.21 (CGEX)) and MST1R gene (rs2230590, OR = 1.03, p = 3.0 × 10^–3^ (CGEX), p = 2.0 × 10^–3^ (PUBB)) has also been associated with neurocognitive function and in our study was associated with IHD in PUBB. However, both DUSP13 and MST1R genes, did not meet statistical significance (p > 3 × 10^–6^) in the CGEX study^27,28^. DUSP13 gene (rs6480771, T/C, C = 57%) missense variant has been predicted to yield protein DUSP13 isoform ([AGC]Ser160Cys[GGC]) (Table 3). A more recent study by Wang et al., using GWAS summary and proteomics data from Medical Research Council Integrative Epidemiology Unit, suggested strong evidence of association of protein-coding regions in DUSP13 with atrial fibrillation^36^. In the same study, using multivariable mendelian randomization analysis, CAD phenotype played a causal role for atrial fibrillation, suggesting shared genetic pathways between atrial fibrillation and CAD^36^. In addition, an-invitro study by Shen et al. demonstrated upregulation of DUSP13 genes in cardiac myocytes in response to cadmium-induced cardiotoxicity^37^. In-vitro studies demonstrating the upregulation of MYBPHL and DUSP13 to cardiac stress and our gene-based analysis observations suggest their role in CAD pathophysiology^34,37^.

Among the top one percent of VEGAS2 genes in CGEX that did not meet our Bonferroni-corrected significance threshold (genes in Table 2 with p > 3 × 10^–6^), KCNJ11(rs5215, OR = 1.03, 1.9 × 10^–4^ (CGEX)) and RAB37 (rs35489971, OR = 1.05, 6.5 × 10^–5^ (CGEX)) have been previously associated with islet cell hyperplasia and fibrinogen levels respectively^38,39^. Both conferred an increased risk of CAD in our study. SNVs in QSER1 and UBR2 has been previously linked with neurodegenerative (Parkinson’s) disease and obesity, respectively^40,41^. In our study, SNVs in QSER1 and UBR2 conferred a higher risk of CAD with a SIFT score lower than the threshold level (P ≤ 0.05), suggesting a high probability of protein function alteration from the amino acid sequence change (Table 3). More recently, Hartiala et al. identified the SNV (rs62618693) in QSER1 through single SNV analysis to be associated with CAD in the UK Biobank cohort, but it failed to reach statistical significance. QSER1 is one of the 17 genes we identified using both GBA methods and in both GWAS studies.

GPA provides insight into the functional implications of enriched genes and the role of different pathways in CAD susceptibility. Through VEGAS2 and MAGMA GPA, we were able to identify the association of neuronal aging/apoptosis, coagulation cascade, inflammation, wound healing in addition to lipid metabolism to be associated CAD. Van der Harst et al. reported coagulation and inflammation pathways using proprietary Ingenuity software in CGEX and PUBB cohorts^6^. Nelson et al. used 300 SNVs associated with CAD to identify pathways related to angiogenesis using Ingenuity software^29^. However, using open source VEGAS2 and MAGMA, we identified the role of neurocognition in CAD and the role of neuronal apoptosis/aging and coagulation cascade for CAD susceptibility. While CAD and vascular dysfunction has long been viewed as a risk factor for cognitive decline, our study highlights the hypothesis of “neurocognitive health” and “neuronal aging/apoptosis” as essential factors modulating CAD risk, likely through shared gene-pathways^12,42–45^. A recent study by Gu et al. noted a high incidence of cognitive decline in older patients presenting with myocardial infarction^46^. More recently, Li et al. demonstrated the association of genetic components of intelligence associated with CAD with an inverse correlation behavioral risk factors of CAD^47^. It has been hypothesized that failure of the glymphatic system leading to aggregation of neurotoxic proteins could be an underlying mechanism for dementia^45^. Glymphatic failure has indeed been linked with cardiovascular disease^48^. Mouse models of Alzheimer’s disease have demonstrated the extracranial aggregation of neurotoxic proteins through the glial-lymphatic system^49^. Our observation of genes linked with neurotoxic tau proteins, and neuronal aging-based pathways associated with CAD raises the possibility of glymphatic system’s role in CAD through extracranial aggregation neurotoxic proteins.

Our study has certain limitations. First, we used publicly available GWAS summary data from the CGEX exome array and PUBB data and limited our analysis only to non-synonymous SNVs. Our approach of using non-synonymous SNVs has been applied in other complex disease GBA and individual SNV association studies.^1,50,51^ When we included all non-synonymous SNVs (rare and common) in our analysis,we did not find any evidence of systemic inflation of p-values. For VEGAS2 GBA, we considered SNVs in the ‘0kbloc’ region, with respect to 5’ and 3’ UTR. This step can reduce the power of detecting regulatory SNVs that are otherwise not tagged by the gene boundaries. However, the inclusion of multiple unassociated SNVs might reduce the power of gene-based approaches. Hence, this step decreased the burden of multiple testing. Moreover, based on the included SNVs, we had sufficient SNV information covering almost 83% of the gene space across all designated NCBI 37.3 autosomal genes. Nevertheless, there remains significant variation in defining gene boundaries across GBA, and any gene-based study should be viewed as a complement to single SNV association studies^52–54^. While not exhaustive, VEGAS2 GPA used canonical pathways and gene-sets from BIOCARTA, REACTOME, PANTHER, gene ontology, pathway commons, and pathway interaction database. In this step, we may have missed other pathway annotation databases that would have otherwise identified novel CAD associated pathways. Our discovery cohort was primarily in individuals from Western European ancestry. However, we were able to replicate many of our findings in multi-ancestry though predominant Western European UK Biobank cohort. In addition, some genes discovered in our study included SNVs have been previously associated with CAD individuals of East and South Asian ancestry^5,55^. We only used SIFT as a tool to evaluate the likely protein function consequences from amino-acid substitutions. We acknowledge that many protein function prediction tools are available using coding SNVs (PolyPhen, SIFT, Grantham, MutationTaster) and some tools like CADD (Combined Annotation Dependent Depletion) that could combine these individual tools^56^. However, a technical report by Kircher et al. comparing many individual tools noted that SIFT score had the highest discriminatory capacity, followed by PolyPhen for protein level metrics^56^. Finally, all observed VEGAS2 and MAGMA associations need functional in-vitro, in-vivo, and population risk modification studies to confirm their physiological significance.

In conclusion, the VEGAS2 and MAGMA gene and pathway analysis lead to discovering previously unreported genes associated with CAD and could map functional pathways involving the discovered loci. In particular, we were able to confirm the coagulation cascade’s role^12^ and identified neuronal health and neuronal aging as critical gene-based pathways associated with CAD. Many SNVs in the VEGAS2 and MAGMA enriched genes and pathways had subthreshold p-values based on the traditional GWAS significance level and underscore the role of subthreshold SNVs and the genes containing the SNVs in CAD pathophysiology. This study also underscores the multiple pathways associated with CAD and the need for a continued multifaceted approach for CAD prevention.

## Methods

### Study participants

#### CGEX study

We performed GBA and GPA on the publicly available CGEX GWAS summary data. The CGEX summary data includes 120,575 (42,335 cases and 78,240 controls) individuals recruited from 20 studies across Europe and North America^1^. Supplement 1 (I) provides details of individual studies. While there was some heterogeneity in the CAD definition across the study cohorts, the case definition for CAD can be summarized as the presence of one or more of the following: a history of myocardial infarction (MI); the presence of stable or unstable angina; a history of percutaneous coronary intervention (PCI) or coronary artery bypass graft (CABG); at least one epicardial coronary artery stenosis (> 50%) in coronary angiogram; International Classification of Disease (ICD-9 or 10) codes compatible with MI or PCI or CABG or chronic ischemic heart disease; abnormal myocardial stress imaging or died due to CAD. Controls were selected from population-based cohorts who were asymptomatic, generally older than case definition criteria, or did not meet the CAD definition as stated above.

#### PUBB study

We performed a replication analysis of our GBA and GPA using the publicly available PUBB GWAS summary data (https://pan.ukbb.broadinstitute.org). In brief, PUBB prospectively recruited 500,000 consented individuals across different ancestries residing in the UK between the ages 40–69 years from 2005 to 2010 and performed a genotypic and phenotypic evaluation^20^. Data across 7221 phenotypes were prospectively obtained through self-reported questionnaires, ICD codes during clinic visits and hospitalizations, biomarker panel, radiographic studies, and other health data points through electronic medical records from National Health Services or other UK National Registries. We used phenocode 411 (developed based on ICD-9 or 10 representing “Ischemic Heart Disease-IHD”) to access publicly available GWAS summary statistics across 442,574 individuals (43,287 cases, 399,287 controls)^57^. We downloaded the summary association statistics (version 3) from the publicly available amazon cloud link provided within the domain https://pan.ukbb.broadinstitute.org/downloads/index.html (https://pan-ukb-us-east1.s3.amazonaws.com/sumstats_flat_files/phecode-411-both_sexes.tsv.bgz). This phenotype was chosen to match the CGEX study case definition and mapped to the following disease entities: stable or unstable angina, MI, mechanical complications from MI, presence of CABG or PCI, atherosclerotic heart disease, and other acute or chronic ischemic heart disease. The PUBB was a pan-ancestry cohort comprising of individuals representing European (EUR, n = 419,724), Central/South Asian (CSA, n = 8870), African (AFR, n = 6624), East Asian (EAS, n = 2708), Middle Eastern (MID, n = 1593), Admixed American (AMR, n = 979) other (n = 2076) ancestries.

### Genotyping and quality control

#### CGEX study

Details regarding each study's genotyping and sample quality control (QC) procedures have been reported earlier by CGEX investigators^1^. In brief, the CGEX investigators performed genotyping on the Illumina HumanExome BeadChip (v 1.0 or 1.1) or the Illumina OmniExome array (including markers from HumanExome BeadChip) per manufacturer’s protocol. Subsequently, phasing and imputation were performed by CGEX investigators using SHAPE IT, IMPUTE2, MACH, and BIMBAM^8,58^. The accuracy of rare variant genotypes was increased using the zCall algorithm^59^. The CGEX investigators performed sample QC on genotypes before the application of the zCall algorithm. The CGEX investigators used the Hardy–Weinberg exclusion threshold of 1.0 × 10^–5^^1,30^.

#### PUBB study

Details regarding genotyping and sample quality control (QC) procedures have been reported earlier by the PUBB study group^20^. In brief, all PUBB individuals were genotyped using the UK Biobank BiLEVE axiom array and the UK Biobank Axiom array. All sample batches (n = 106) were genotyped at the Affymetrix Research Services Laboratory in Santa Clara, California, USA. The Affymetrix analysis resulted in 812,428 markers (biallelic SNVs and indels) used for QC. QC approach accounted for the large cohort size, batch type processing, and population structure. Specifically, they tested for batch effect, plate effect, a departure from HWE, and sex effect to each marker in each batch. Markers that failed anyone these tests in every batch were excluded. Subsequently, markers failing array effect or had discordance across controls were excluded. This screening led to 805,426 markers across 488,377 samples. Subsequently, allele frequencies were matched across an independent Exome Aggregation Consortium database (ExAC). For imputation, markers with a greater than 5% missing rate across all batches or with MAF < 0.0001 were removed, leading to 670,739 autosomal markers. Imputation was carried out using the Haplotype Reference Consortium. Imputation was carried out using the IMPUTE4 program (https://jmarchini.org/software/). Subsequently, SNVs with INFO (proportion of imputed SNVs equivalent to set of perfectly observed genotypes) score > 0.8 were retained.

### Statistical analysis

#### CGEX GWAS

We utilized the CGEX GWAS summary data (Supplement 1, II) to identify all non-synonymous variants located on the 22 autosomes. For mapping the SNV location to dbSNP rsID, we used “SNPInfo_HumanExome-12v1_rev5.tsv.txt” downloaded from https://chargeconsortium.com/main/exomechip. For categorizing the variants using dbSNP rsID, we used the Annovar tool (http://www.openbioinformatics.org/annovar/). Using dbSNP rsID and corresponding functional annotations, we excluded synonymous, intergenic, intronic SNVs, and SNVs with missing annotations (Fig. 1). To evaluate the association of single SNV with CAD, the CGEX investigators performed logistic regression with additively coded genotypes, CAD as the dependent variable, adjusting for top ten principal components of ancestry, excluded monomorphic SNVs, and combined evidence across studies using an inverse variance weighted fixed-effect meta-analysis. CGEX investigators restricted the meta-analysis of autosomal SNVs with a minor allele frequency of ≥ 0.1% across the 120,575 samples in the discovery cohort. To detect systemic inflation of SNV association p-values, we plotted a quantile–quantile (QQ) plot of observed versus expected p-values from the CGEX GWAS summary data (Supplement 1, FI). Each study was corrected for genomic control prior to assessing SNV association analysis at the GWAS level. Because each study was adjusted for genomic control prior to meta-analysis, we did not adjust GWAS association statistics for genomic control as it may not represent overdispersion due to population stratification but rather represent true genetic signals^21,22^.

#### PUBB GWAS

The PUBB investigators performed GWAS for each phenotype and ancestry group using linear or logistic regression (SAIGE = Scalable and Accurate Implementation of GEneralized mixed model package), including random effects to account for correlated data, as defined by the empirical kinship matrix and covariates as fixed effects. Each GWAS model used age, sex, and the first 10 PCs as covariates. We used the GWAS summary statistics for the phenotype “IHD” and excluded synonymous, intergenic, intronic SNVs. This filtering led to the identification of 140,911 SNVs. We further restricted our analysis to autosomal SNVs with MAF ≥ 0.1% across 442,574 individuals to include autosomal 85,206 SNVs for our replication analysis. To detect systemic inflation of SNV association p-values, we plotted a quantile–quantile (QQ) plot of observed versus expected p-values from the PUBB GWAS summary data (Supplement 2, FI).

### (A) Single SNV analysis

We used Manhattan plots to highlight all SNVs associated with CAD at the significance threshold of p ≤ 1.0 × 10^–4^. We further used SNVs with CAD association p ≤ 10^–4^ to confirm previously reported CAD-associated loci. To omit SNVs with substantial differences in sample size and effect estimates across the 20 studies, we used the Cochran heterogeneity test threshold of p ≤ 0.1. SNVs with non-heterogeneous effects (Cochran heterogeneity test p-value > 0.1) with association p-values below our genome-wide significant threshold (p < 5.0 × 10^–8^) were considered to be significantly associated with CAD in the CGEX data. For replicating single SNV analysis in the PUBB study using 85, 206 SNVs, we defined our type 1 error significance threshold at the Bonferroni corrected value of 5.0 × 10^–7^ ($\frac{0.05}{\mathrm{85,206}})$.

### (B) VEGAS2 GBA

For VEGAS2 GBA, we used the online web server implementation tool (https://vegas2.qimrberghofer.edu.au) to rank genes and pathways. While there is no gold standard practice to define gene boundaries, we considered SNVs in the ‘0kbloc’ region, with respect to 5’ and 3’ UTR (Untranslated Region), to focus on exonic SNVs and excluded regulatory regions^52,60^. Consistent with the software gene boundary options, our method also reduces the problem with the annotation of overlapping genes^61^. Details regarding the VEGAS2 gene and pathway-based analysis have been provided in the study by Mishra et al.^19^ Gene annotation was performed according to NCBI (National Center for Biotechnology Information) build 37/hg 19.

In brief, for gene-based analysis, the p values for n SNVs within the specified gene boundary were converted to an upper tail χ^2^ statistic with one degree of freedom and summed to calculate a gene-based test statistic. The significance of gene-based test statistic was compared to simulated replicates from a multivariate normal distribution with mean = 0 and variance = Σ (the n × n correlation matrix between the SNV genotypes within the gene using LD values estimated from 1000 Genomes European reference population for both CGEX and PUBB). Empirical p values were computed for each gene using the formula, Emp p-value =$\frac{\mathrm{r}+1}{\mathrm{m}+1}$, where r is the number of instances where the simulated statistic exceeds the observed data and m is the number of simulations (starting at 1000 simulation replicates and progressively increasing the number of simulation replicates to 10,000 for genes with p < 0.1, and to 100,000 for genes with p < 0.01, and to 1 million simulation replications for genes with p < 0.001). An r of 0 from 10^6^ simulations can be interpreted as p < 10^–6^, which exceeds the Bonferroni-corrected threshold of 3 × 10^–6^ ($\frac{0.05}{\mathrm{15,296}}$) for genes. In the CGEX study, a total of 4796 genes (SNVs > 1 per gene) in the ‘0kbloc’ gene boundary had empirical p-value estimates < 1; the remaining 10,500 genes had VEGAS2 empirical p-values exactly equal to 1 or had 0 or 1 SNVs in the gene boundary. In the PUBB study, a total of 6096 genes (SNVs > 1 per gene) in the ‘0kbloc’ gene boundary had empirical p-value estimates < 1.

In addition to genes meeting our Bonferroni-corrected threshold of 3 × 10^–6^ for genes, we also investigated the top one percent of genes with CAD association empirical p-values < 1, as a suggestive threshold (enriched genes). Our type-1 error significance threshold definition is consistent with other GBA studies^5,62^. We also performed Manhattan plot analysis to highlight all enriched genes identified through VEGAS2 GBA.

### (C) MAGMA GBA

For MAGMA GBA, we used MAGMA v1.08b obtained from https://ctg.cncr.nl/software/magma for our analysis. We further downloaded NCBI build 37.3 to map non-synonymous SNV to 15,400 genes from the total 18,575 autosomal NCBI 37.3 gene list using our gene boundary definition (0kbloc). We subsequently downloaded 1000 Genomes European panels that MAGMA uses as reference data to account for LD between SNVs and compute the correlation matrix for SNV genotypes. For our analysis, we used the default SNP-wise mean model, where a T statistic is calculated from the sum of squared SNV Z-statistics (T^*^ = ${\sum }_{\mathrm{j}}^{\mathrm{K}}{\mathrm{Z}}_{\mathrm{j}}^{2}={\mathrm{Z}}^{\mathrm{T}}\mathrm{Z}$, *Z*j = Φ(p_j_), p_j_ = marginal p-value for SNV j). Z is assumed to have a multivariate normal distribution with mean = 0 and variance = the n × n correlation matrix between the SNV genotypes within the gene using LD values estimated from 1000 Genomes European reference population. This summed statistic is used for calculating gene-based p values^18^. In the CGEX study, the MAGMA GBA list included 10,029 genes with more than one SNVs. In the PUBB study, the MAGMA GBA list included 10,195 genes with more than one SNVs. We defined our type-1 error cut off after Bonferroni correction at 3 × 10^–6^ ($\frac{0.05}{\mathrm{15,400}}$) based on the total mapped NCBI build 37.3 mapped genes. In addition to genes meeting our Bonferroni-corrected threshold of 3 × 10^–6^ for genes, we also investigated the top one percent of genes with CAD association empirical p-values < 1, as a suggestive threshold (enriched genes).

### (D) Protein function and clinical phenotypic significance of top SNVs in identified genes

For the top enriched genes identified by VEGAS2 with previously unreported CAD associations, we recorded the previously reported clinical phenotypes using the Ensembl genome database (https://www.ensembl.org), the National Center for Biotechnology ClinVar web-based database (https://www.ncbi.nlm.nih.gov/clinvar/variation/), and through Pubmed central search (https://pubmed.ncbi.nlm.nih.gov). We further used the “Sorting Tolerant From Intolerant (SIFT)” tool to identify protein-altering functional significance of coding SNVs among the top enriched genes^63^. SIFT predicts protein function alteration from the amino acid substitution, based on a scaled probability threshold, also known as the SIFT score. SIFT score ranges from 0–1, and score ≤ 0.05 suggests protein alterating functional consequence. However, SIFT does not account for dynamic protein structural changes from amino acid sequence change that could affect protein function.

### (E) VEGAS2 GPA

VEGAS2 uses gene ontology incorporated from BIOCARTA, REACTOME, PANTHER, pathway commons, and pathway interaction database for pathway-based analysis^53,64–67^. We defined non-lipid based pathways as those without the terms “lipid or lipoprotein or lipase or sterol or triglyceride or cholesterol” in the pathway names. VEGAS2 computes pathway-based summed chi-squared (${x}^{2}$) statistics, by converting gene-based p-values to upper tailed χ^2^ statistics with one degree of freedom before summing. While the gene p-value was obtained from the summed chi-squared (${x}^{2}$) statistic with degrees of freedom equal to the number of SNVs in the gene, the empirical p-value for pathway was calculated by repeatedly resampling the same number of genes drawn at random from the pathway under consideration. Empirical p-value was defined as p =$\frac{y+1}{N + 1}$ , where y is the number of instances the summed chi-squared (${x}^{2}$) statistic per resample is more than or equal to the observed for pathway under consideration and N is the number of resamples performed^53^. The resampling approach corrects for varied pathway sizes. Pathways were ranked according to empirical p values. From the ranked genes, the VEGAS2 GPA ranked 5528 pathways that had empirical p-values < 1 in the CGEX study. Despite the non-independent nature of the gene pathways, we used a rigorous Bonferroni-corrected pathway significance threshold of 10^–5^ ($\frac{0.05}{5528}$). For the PUBB study, we used a rigorous Bonferroni-corrected pathway significance threshold of 10^–5^ ($\frac{0.05}{5764})$, based on the ranked pathways with empirical p values < 1. In addition to reporting pathways meeting our rigorous Bonferroni threshold, we also investigated the suggested threshold of the top 1% pathways associated with CAD with empirical p-value estimates < 1.

### (F) MAGMA GPA

For GPA, MAGMA transforms the gene-based p-values to standard normal Z statistic with lower p-values corresponding to higher Z statistic. Gene pathway analysis is implemented using a linear regression model, where the gene association score (Z statistic) is tested for association with gene pathway membership (S), adjusting for gene-level covariates (C): *Z* = β_0_ + *S*β*s* + *C*β*c* + ε. The error term (ε) is assumed to follow a multivariate normal distribution with correlation matrix computed from the gene–gene correlation obtained from the gene analysis resampling. For pathway annotation, we used the Biosystems pathway containing 9574 gene pathways. We defined our type-1 error cut off after Bonferroni correction at 5 × 10^–6^ ($\frac{0.05}{9574}$). We further investigated the top 1% of listed pathways as a suggestive threshold.

### (G) VEGAS2 and MAGMA comparison

We tabulated a list of genes meeting our significance threshold (3 × 10^–6^) in CGEX and PUBB, respectively. In addition, we constructed a scatter plot comparing ranks of top 1000 genes common to VEGAS2 GBA and MAGMA GBA across CGEX and PUBB, respectively, and computed Spearman rank correlation. We further plotted a Venn diagram investigating the overlap of the top one percent of listed genes identified by both GBA methods in the CGEX and PUBB. For pathway comparison, while different pathway annotation sets were used for each GPA method, we categorized pathways into lipid and non-lipid pathways and compared the top 1% of ranked pathways. We used R version 4.0.3 for the Venn diagram, scatter plot and single SNV analysis. For SNVs screened after VEGAS2 and MAGMA analysis we obtained LD information from NCBI LD calculator tool (https://ldlink.nci.nih.gov/?tab=home) to identify previously CAD-associated SNVs in LD with our screened SNVs. We defined SNVs in high LD, if r^2^ > 0.8 within ± 500 kb distance from our screened SNV location.

## Supplementary Information

Supplementary Dataset 1.

Supplementary Dataset 2.

## Data Availability

All data generated or analyzed during this study are included in this published article (and its Supplementary Information files). Supplemental Materials 1 and 2; Expanded Materials & Methods; Data Set; Online Figures I–II.

## Abbreviations

GWAS: Genome wide association studies
CAD: Coronary artery disease
SNV: Single nucleotide variants
CGEX: CARDiOGRAM exome studies
PUBB: Pan UK biobank
GBA: Gene based association analysis
GPA: Gene pathway association analysis
VEGAS: Versatile gene based association studies
MAGMA: Multi-marker analysis of genomic annotation
SIFT: Sorting tolerant from intolerant
